# “It's okay to feel!”: How a music‐based pedagogical activity fosters medical students' emotional development

**DOI:** 10.1111/medu.70073

**Published:** 2025-11-03

**Authors:** Marcelo B. S. Rivas, Agnes F. P. Cruvinel, Daniele P. Sacardo, Daniel U. C. Schubert, Mariana Bteshe, Marco A. Carvalho‐Filho

**Affiliations:** ^1^ Medical Emergencies Department Rio de Janeiro State University ‐ UERJ Rio de Janeiro RJ Brazil; ^2^ Bauru Medical School University of São Paulo ‐ FMBRU/USP Bauru SP Brazil; ^3^ Public Health Department Campinas State University Campinas SP Brazil; ^4^ Medical Psychology Rio de Janeiro State University ‐ UERJ Rio de Janeiro RJ Brazil; ^5^ Wenckebach Institute for Education and Training, Lifelong Learning, Education and Assessment Research Network (LEARN) University Medical Center Groningen (UMCG) The Netherlands

## Abstract

**Background:**

Emotions are an intrinsic part of medicine. However, formal medical curricula fall short in addressing the role of emotions in medicine, and the hidden curriculum often promotes emotional detachment as a core component of medical professionalism. In this study, we addressed the following research question: what are the mechanisms through which a music‐based pedagogy grounded in emotion regulation (EmtR) nurtures medical students' emotional development?

**Method:**

In this cross‐sectional, qualitative study, we performed a reflexive thematic analysis with an inductive approach grounded in the constructionist paradigm. The pedagogical activity comprehended four encounters, and music listening sessions were used to evoke emotions. The encounters were conceptualized to address emotion expression, identification, regulation and the impact of emotions in clinical care. We recruited 25 participants (21 students and 4 facilitators) from three Brazilian medical schools who took part in semi‐structured interviews in 2020 and 2021.

**Results:**

Our analysis resulted in four co‐constructed themes explaining the mechanisms through which the music‐based pedagogical activity nurtured students' emotional development: (a) **Creating a safe and pleasant environment** – music listening facilitated emotional expression in a safe, democratic and supportive environment; (b) **Facilitating Emotional Connections** – shared emotional experiences during collective music listening strengthened connections among students and facilitators, showing how the same experience may evoke different emotional responses; (c) **Providing opportunities to engage with EmtR strategies** – students reflected on the impact of emotions on their personal and professional development, experiencing and simulating different EmtR mechanisms; and (d) **Naturalizing Emotions in Medicine** – students reported that music facilitated reflection on the role of emotions in medicine and helped them integrate their emotional selves into their professional roles, valuing emotions as essential to being a doctor.

**Conclusions:**

This study clarifies the mechanisms through which music‐based pedagogical interventions can nurture medical students' emotional development, contributing to a broader understanding of how the arts may counteract the culture of emotional detachment in medicine.

## Abstract 2

Emotions matter in medicine. Our study shows how a music‐based pedagogy can support medical students' emotional development and challenge the culture of emotional detachment. #MedEd #ArtsInMedicine

## 1 INTRODUCTION

Emotions are at the core of medical practice, and the journey to become a doctor is deeply interwoven with emotional experiences.^1^, ^2^ During this journey, medical students who effectively manage their emotions not only forge stronger emotional connections with their patients but also exhibit a lower risk of mental illness.^3^, ^4^ Regrettably, medical schools dedicate little space in the curricula to nurture the emotional development of medical students and discuss the importance of emotions in medical practice, which may inadvertently lead students to compartmentalize and detach from emotions, a response that risks triggering depersonalization and cynicism.^4^, ^5^, ^6^ To counterbalance this trend, medical educators have introduced a myriad of educational practices.^7^, ^8^ In alignment with this endeavour, we developed and implemented an innovative pedagogical activity based on emotion regulation (EmtR) concepts and music to nurture the emotional development of medical students.^9^ In this qualitative study, we investigated the mechanisms through which a music‐based pedagogical activity supported medical students' emotional development. By exploring these mechanisms, we aim to contribute to the ongoing conversation about the role of the arts, particularly music, in emotional development.

We understand emotional development as “the emergence of the experience, expression, understanding, and regulation of emotions from birth and the growth and change in these capacities throughout childhood, adolescence, and adulthood”.^10^ Emotional development occurs in conjunction with neural, cognitive and behavioural development and emerges within a particular social and cultural context.^10^ Addressing medical students' emotional development is relevant because during medical training and practice, they must navigate and manage a variety of complex emotional encounters, permeated by intense emotions like shame, anger, disgust and solitude.^6^, ^11^, ^12^, ^13^, ^14^ However, medical students often graduate feeling unprepared to acknowledge patients' emotions and also with difficulties in recognizing, expressing and regulating emotions.^6^, ^15^ This difficulty in managing emotions related to medical practice may lead to emotional detachment and cynicism and is associated with students' burnout.^16^, ^17^ Besides causing psychological distress for the student, this emotional detachment also affects patients, who feel frustrated at not having their emotions addressed.^18^ Conversely, medical students' emotional development positively correlates with some core competencies, such as communication skills, professionalism, coping with work pressure and delivering empathetic patient care.^19^

Although medical educators have recognized the importance of fostering the emotional development of medical students, they still struggle to identify a conceptual framework to guide the design and implementation of tailored educational interventions.^20^ One of the possible frameworks to inform this educational design is Emotion Regulation (EmtR). EmtR was introduced in psychology three decades ago, and it is defined as “the processes by which individuals influence which emotions they have, when they have them, and how they experience and express these emotions.”^21^, ^22^ According to EmtR, emotions may be regulated by five sets of regulatory strategies: (a) situation selection (choosing or avoiding situations based on the anticipated emotional outcomes); (b) situation modification (modifying aspects of their environment to change its emotional impact); (c) attentional deployment (redirecting attention within a given situation to influence emotional responses); (d) cognitive reappraisal (reframing or reappraising situations in a way that alters their emotional significance); and (e) response modulation (modifying physiological, emotional or behavioural responses to regulate emotions).^21^ EmtR is a multifaceted process influenced by a complex interplay of biological, social, psychological and environmental factors.^23^, ^24^ By employing EmtR strategies, individuals can modulate the intensity and duration of their emotional responses, aiming to better adapt and navigate emotionally challenging situations. Currently, there is no consensus regarding the best approach to develop EmtR in medical students, but research shows a great potential for art‐based pedagogical activities, including music.^25^

Music is a universal language that plays an important role in education due to its substantial influence on individual physiological, social, cognitive and emotional dimensions.^26^, ^27^ Music and emotions coalesce naturally^28^ and although most studies have traditionally focused on social aspects of music, recent data suggest that one of the fundamental functions of music is EmtR.^27^, ^29^ EmtR by music encompasses the elicitation and modulation of emotional responses, the retrieval of autobiographical memories and the induction of physiological changes that broaden emotional awareness. We believe that wedding this broadened emotional awareness evoked by music, with the application of the EmtR concepts in a pedagogical activity, is a way to trigger a reflective process about the role of emotions in medical practice.^9^, ^30^, ^31^, ^32^

In a previous innovation report, we described the implementation of a music‐based pedagogical activity to nurture the emotional development of medical students from three Brazilian medical schools.^9^ This report focused on describing the activity and adopted a quantitative, pre‐post design using the Schutte Self‐Report Emotional Intelligence Test (SSEIT) to assess changes in students' emotional intelligence levels following participation in the activity.^9^ However, during the activities, we understood that a quantitative analysis of students' emotional intelligence lacked nuance to explore and understand the mechanisms through which the activity supported students' emotional development.^9^ Thus, to deepen our understanding, in this qualitative study, we examined the following research question: what are the mechanisms through which a music‐based pedagogy nurtures medical students' emotional development? By qualitatively exploring this question, we aim to provide meaningful insights that may inform and contribute to the ongoing dialogue about the role of arts in medical education.

## 2 METHODOLOGY

### 2.1 Study design

In the current cross‐sectional qualitative study, we explored participants' subjective accounts to identify the mechanisms through which a music‐based pedagogy nurtures medical students' emotional development. Our reflexive thematic analysis followed an inductive logic, consistent with a constructionist epistemology, recognizing that emotional development is not a universal or static process but is shaped by individual narratives and interpersonal dynamic connections. We analysed semi‐structured interviews with medical students and facilitators who joined this activity. Although the previous innovation report included all the 79 students who completed the SSEIT at pre‐ and post‐intervention, in this present study, we interviewed a purposeful sample of 21 students and four facilitators recruited from three Brazilian public universities, who participated in one‐off, individual semi‐structured interviews. We aimed to capture participants' meaning‐making processes and subjective experiences, offering insights into the mechanisms through which the pedagogical activity, grounded in music and EmtR, functioned to foster medical students' emotional development.

#### 2.1.1 The music‐based pedagogical activity

The activity “Emotions in Medicine” consisted of four 2‐hour sessions conducted at three Brazilian medical schools: Rio de Janeiro State University (UERJ – site 1), Federal University of Fronteira Sul (UFFS – site 2), and State University of Campinas (UNICAMP ‐ site 3). The three sites were selected based on convenience, as the first author (MR) is a faculty member at site 1, and two coauthors are affiliated with site 2 (AC) and Site 3 (DPS), respectively. The activity was offered in person as an optional summer course for fourth‐year students in January 2020. At sites 2 and 3, it was integrated into the third‐year medical curriculum and delivered online due to the COVID‐19 pandemic, in May and July 2021, respectively. In Brazil, the medical course has 6 years, divided into three periods of two years: basic sciences, pre‐clinical and clinical. However, students have contact with patients and the healthcare system since the first year on a regular basis.

**FIGURE 1:**
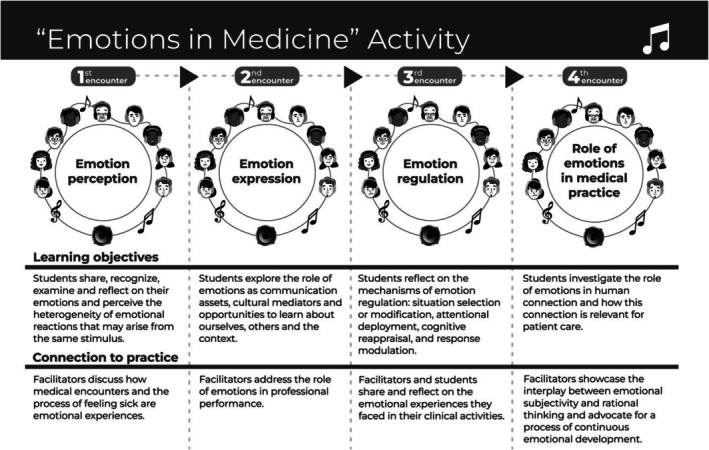
Visual representation of the four encounters making up the music‐based pedagogical activity “Emotions in Medicine.” Reproduced with permission from Rivas MBS, Cruvinel AFC, Sacardo DP, Schubert DUC, Bteshe M, de Carvalho‐Filho MA. All You Need Is Music: Supporting Medical Students' Emotional Development With a Music‐Based Pedagogy. Acad Med. 2024;99(7):741–744. doi:10.1097/ACM.0000000000005709.

Each of the four sessions addressed different dimensions of emotional development: emotion expression, identification, regulation and the role of emotions in medical practice. The activity had four sessions with similar structures and combined music listening, facilitated group discussions and clinical vignettes to stimulate the exploration of EmtR mechanisms. First, students listened to pre‐selected songs and shared their personal impressions. Next, students reflected on their emotional status. After investigating and understanding their emotional selves, students reflected on clinical situations charged with different emotional components. Finally, students and facilitators shared and reflected on personal clinical experiences that were particularly emotional. Figure 1 synthesizes the learning objectives and the practical implications of the four sessions of the activity “Emotions in Medicine”. Box 1 displays examples of exercises combining music listening, facilitated group discussions and clinical vignettes used in the sessions of the activity “Emotions in Medicine”.

Box 1 **Examples of exercises combining music listening, facilitated group discussions and clinical vignettes used in the sessions of the activity “Emotions in Medicine”.**

**Exercise used in session 3 to stimulate the exploration of EmtR mechanisms.**

The facilitators played a song about a father mourning his son's suicide together with the singer, a friend of the deceased. The song metaphorically describes how they drank together a pure, crystal‐clear drink, shedding “crystalline tears” together and finding solace through cognitive reappraisal, ultimately realizing that the son's death, a consequence of treatment‐resistant depression, was not their fault. Following this music listening session, a clinical vignette about a 40‐year‐old woman rushed by her teenage daughter to the hospital in cardiac arrest was presented. Despite resuscitation efforts, the woman died, and her daughter became overwhelmed with guilt. In the discussion, students were invited to reflect on how they could support the daughter through cognitive reappraisal, helping her understand the mother's underlying cardiac risks and the exhaustive measures taken trying to save her.

**Exercise used in session 4 to illustrate the role of emotions in medical practice.**

The facilitators played a song where the students listened to an emotionally charged singer who cried during his performance of a sad song. Crying reduced his vocal range; nevertheless, the audience in the video recognized and valued the intense emotion transmitted by the singer when he exposed his true emotion, which created a unique connection with the audience in an outstanding performance. Facilitators used this example to underscore emotions as an important element of professional life by inviting students to reflect on how a genuine expression also led to an increase in their appreciation for the performance of this artist. Inspired by this perception of the value of emotions in professional life, facilitators shared with the students that it is possible to have meaningful emotional experiences during their clinical practice. In the subsequent discussion, students and facilitators shared examples from clinical settings where physicians, while visibly emotional, demonstrated compassion and relational presence, particularly during emotionally demanding tasks such as breaking bad news. These reflections helped deconstruct the notion that emotions undermine clinical competence, encouraging students to recognize emotional expression as both inevitable and potentially constructive in establishing meaningful doctor–patient relationships.

A total of eight trained facilitators familiar with EmtR theory were involved in the activity. At sites 1 and 2, the sessions were facilitated by the main author with one or two local facilitators. At site 3, the main author trained four independent facilitators to explore the reproducibility of the activity.

### 2.2 Participants and ethical approval

We adopted a purposeful sampling strategy to ensure representation of students from the three participating sites and to include facilitators with diverse professional backgrounds. All students from site 1 (n = 8) and site 2 (n = 13) who participated in the pedagogical activity were invited to take part in the qualitative study. All students at site 1 and most at site 2 (n = 9) agreed to be interviewed, resulting in a sample sufficiently diverse in terms of gender and cultural backgrounds. At site 3, where a larger group participated in the activity (n = 58), a smaller subset (n = 4) was purposefully selected for interviews based on their perceived high level of engagement during the sessions. Facilitators were selected based on their distinct professional backgrounds, including a junior doctor, a nurse who is also a lawyer, a psychologist and a senior physician. This sampling approach aligns with qualitative research methods that aim to capture a range of perspectives by emphasizing maximum variation through the inclusion of participants from diverse backgrounds. Such variation helps to uncover complexity and prevents the findings from being overly narrow.^33^, ^34^

Students and facilitators were approached for the interviews in person, by telephone messaging or e‐mail by a member of the research team. Students or facilitators who decided to participate contacted the first author to schedule the interview. Participants were informed that the data would be anonymized and treated confidentially, that participation was voluntary and that they could withdraw at any time. All participants gave written informed consent, and only the first author had access to participants' identities and audio tapes. The demographic characteristics of all students and facilitators interviewed are presented in Table 1. Ethical approval was granted by the Research Ethics Committee on Human Beings at the universities (STUDY#3.276.206 in 04/22/2019, STUDY#4.626.426 in 04/02/2021 and STUDY#4.605.559 in 03/22/2021).

**TABLE 1:** Sociodemographic Characteristics of the Participants.

Participants	Age	Gender	Site	College Year
Facilitator 1	40	Female	1	‐
Facilitator 2	26	Male	1	‐
Facilitator 3	39	Male	3	‐
Facilitator 4	33	Female	3	‐
Student 1	29	Female	3	3rd
Student 2	22	Male	3	3rd
Student 3	23	Female	3	3rd
Student 4	24	Female	3	3rd
Student 5	24	Female	2	3rd
Student 6	22	Female	2	3rd
Student 7	23	Female	2	3rd
Student 8	22	Female	2	3rd
Student 9	23	Female	2	3rd
Student 10	22	Female	2	3rd
Student 11	33	Male	2	3rd
Student 12	22	Female	2	3rd
Student 13	22	Female	2	3rd
Student 14	23	Male	1	4th
Student 15	29	Female	1	4th
Student 16	24	Female	1	4th
Student 17	22	Male	1	4th
Student 18	27	Female	1	4th
Student 19	26	Male	1	4th
Student 20	25	Female	1	4th
Student 21	25	Male	1	4th

Note *N*: 25.

### 2.3 Data collection

Student‐participants' average age was 24.4 (SD = 2.9) years, and 15 (71.4%) were female. Facilitator‐participants' average age was 34.5 (SD = 6.4) years, and 2 (50%) were female. The interviews lasted from 22 to 69 min, with an average duration of 32.2 min. Ten interviews (eight students and two facilitators) were conducted in person at site 1 in February and March 2020, prior to the COVID‐19 lockdown. The remaining 15 interviews were conducted remotely via the Zoom platform between June and August 2021, with participants from sites 2 and 3. The activity took place in January 2020 at site 1, in May 2021 at site 2 and in June 2021 at site 3. Across all sites, interviews were conducted within approximately 30 to 60 days following the conclusion of the activity, allowing participants to reflect on their experiences. The interviews focused on understanding the mechanisms through which the activity fostered emotional development, by exploring participants' experiences with (a) listening to music and perceiving emotions in a medical classroom; (b) sharing emotions related to personal and professional issues; (c) engaging with EmtR mechanisms during the activity; and (d) making sense of the role of emotions in medical practice. In the quotes presented in the results section, participants were identified using a code that includes their role (S for student; F for facilitator), a numerical identifier (1–21 for students; 1–4 for facilitators), gender (M: male; F: female) and site (1, 2 or 3). For example, “S7‐F‐U2” refers to student number 7, female, from site 2.

### 2.4 Data analysis

We carried out a qualitative reflexive thematic analysis with an inductive approach grounded in the constructionist paradigm using an iterative process of data collection and analysis.^35^, ^36^ The interviewer followed a semi‐structured guide but remained open to exploring new topics that arose during the interview. Initial insights from the first set of interviews guided subsequent data gathering. The interviews were transcribed verbatim, and all members of the research team participated in the qualitative analysis. MR and MACF were responsible for coding the interviews, continuously refining the process through regular weekly meetings. In these weekly meetings, MR and MACF deepened their analysis, reorganizing, comparing and integrating codes to generate themes. After coding the first five interviews, MR and MACF sent the same interviews to be coded by DPS, MB, DUCS and AC to enrich the meaning‐making process with different perspectives.

MR created thematic maps to serve as visual tools to structure the data, which allowed for the clustering of codes and the construction of overarching themes. MR shared and discussed the thematic maps with MACF to consolidate a mutual understanding of the data, revisiting the original data to increase the rigour. MR kept logbooks of the interviews and meetings to track and make sense of insights and understandings. The analysis culminated in the writing of the report, which allowed for the development of final insights. The report thus served not only as a documentation of the research findings but also as a platform to integrate the thematic analysis and actively engage the entire research group in the consolidation of our understanding.

Relevant quotations were selected from the interviews to exemplify and substantiate the themes identified in the analysis, ensuring that the participants' voices were authentically represented.

### 2.5 Research group and reflexivity

MR is a cardiologist and professor of medical emergencies with over two decades of clinical experience and a strong interest in the role of emotions in medical practice. His passion for the arts, particularly music, has informed his personal and professional journey; he is an amateur guitarist and composer. Considering the impact that music has on emotions, this background in music likely heightened his sensitivity to make sense of the emotional experiences of both patients and healthcare professionals. AC is a speech‐language pathologist who teaches communication skills and incorporates listening to music in her classes. She used to play the keyboard and practice dance modalities (ballet, contemporary dance and jazz). MB is a psychologist who, beyond teaching medical psychology, integrates the student psycho‐pedagogical support program team at one of the institutions, offering mental health support for medical students. She also appreciates music and is an amateur drummer. DUCS, an emergency medicine resident and music admirer, offered valuable insights into musical preferences and emotional reactions within his generation. DPS is a psychologist who teaches in the field of ethics and is an enthusiast of emotional development research, with a deep admiration for music. MACF is a full professor of health profession education at the University of Groningen and has wide experience with qualitative research projects and educational innovations involving arts (theatre, rich pictures, music) and is also a music connoisseur – he is an amateur percussionist. He also incorporated music in many of his classes with the aim of evoking emotions, taking advantage of music's metaphorical nature to facilitate deeper reflections.

The research team's interest in music, combined with its belief in the crucial role of emotional development for integrated medical practice, significantly influenced the design of the educational intervention and the research process. Their background allowed for a nuanced understanding of the performers, their biographies and the musical elements within the lyrics and compositions, which enriched the meaning‐making processes and facilitated the generation of relevant themes from the interviews.

## 3 RESULTS

Our data analysis led to the co‐construction of four main themes that highlight the mechanisms through which the music‐based pedagogical activity nurtured medical students' emotional development. The following paragraphs explore how the activity created a feeling of safety that facilitated emotional connections, allowing students to experience EmtR mechanisms, manage their emotions and understand emotions as a natural constituent of medical practice. When presenting the themes, we use the word “participants” to refer to students and facilitators. Otherwise, the two groups are mentioned specifically.

### 3.1 Creating a safe and pleasant environment

During the sessions, music listening and emotional sharing functioned as mechanisms to create a safe and supportive environment. Although expressing their emotions, students witnessed the emotional reactions of the facilitators, who had the courage to express their vulnerabilities, which functioned as an opportunity for students to feel comfortable in embracing their own vulnerability. This capacity of students and facilitators to feel and be vulnerable may have contributed to the co‐creation of a democratic and horizontal space. Some students perceived how valuable their emotions were to the activity and actively engaged in the discussions, with a feeling of reward, purpose and curiosity about each other's emotions.

“We had the opportunity to listen to songs suggested by our colleagues, so we ended up getting in touch with some musical interests and songs that hold a lot of meaning for other people. I thought it was nice—I found it pleasant.” S15‐M‐U2

Some students reported that the safe environment enabled the release of emotions often repressed during their daily academic routine. For example, one student described her experience as akin to emerging from a “cocoon”, because she was able for the first time to express intense emotions related to the challenges of the medical course. Supported by her peers and the facilitator, she felt encouraged to share her fear of not meeting patients' expectations, an emotion that had been tormenting her. The mutual emotional engagement of students and facilitators, incited by music, contributed to create trust, which favoured further emotional sharing and nourished a virtuous cycle of emotional bonding.

“It's like I'm in a cocoon and this is an opportunity for me to expand, you know? So, this is helping me a lot.” S11‐F‐U2

Besides feeling safe, some students reported that the integration of music listening into the classroom setting functioned as a mechanism to create a relaxing environment, by providing a rare opportunity to slow down and escape from an overall busy medical curriculum. The relief from the tensions of the medical training associated with the possibility of venting their repressed emotions seems to have generated a pleasant experience. They often evaluated the activity as an opportunity to “take a breath” and “connect body and mind”. The combination of the safe environment, the expression of repressed emotions and the contrast with the arduous routine of the medical curriculum created conditions for students to value and engage with the activities.

“Like a moment to take a breath. I thought it was pleasant. It was something very different. Very dissonant from the rest of what we were doing at the time. Dissonant in a good way.” S18‐F‐U3

### 3.2 Facilitating emotional connections

Participants reflected on how feeling safe facilitated the creation of a sense of intimacy among them. When sharing their emotional reactions to certain songs, participants also explained why these reactions occurred and how. These rich emotional narratives involved memories, milestones and lived experiences related to participants' biographies. Most participants pondered that exploring each other's biographies was a powerful and rewarding experience that generated an increased sense of belonging. This sense of belonging motivated some students to seek more emotional encounters inside and outside the classroom. These encounters offered the opportunity for students to engage in meaningful relationships with their colleagues and further nurture emotional connections.

“When we listened to each other's music, I think it was really beautiful to be able to get to know a bit more about each person, even if it was a song that I didn't listen to on my own. I realized: wow, this song could be cool, you know? I think this applies to human relationships, to other subjects we study. So, it certainly created a greater connection.” S13‐F‐U2

Music listening functioned as a catalyst for the recognition of multiple emotional perspectives. Although connecting with their own emotional selves, some students felt compelled to identify and explore other emotional realities. These students realized that a certain stimulus (in this case, music) may evoke different emotional responses depending on the context and individuals involved. These often contradictory emotional reactions aroused curiosity in some students, who took the opportunity to discover distinct perspectives, expanding their capacity to make sense of and relate to others.

“I think my opinion was more about the plurality that music and art bring to us. To see a friend's point of view, which had never crossed my mind, I'd never thought about it, and to see that there is really a connection. To better understand the way the other person felt, which is totally different from how I felt. And that it also makes sense to feel that way.” S6‐F‐U1

Often, this curiosity to explore evolved into a curiosity to imagine and experience these new emotional responses, not only by observing their colleagues` emotions, but also by letting themselves go in a process of emotional contagion during the sessions. Some participants believe that the exercise of imagining unfamiliar emotional realities may have enabled them to incorporate these newfound emotional responses into their emotional repertoires. In this sense, listening to music and exploring the emotional realm of peers functioned as a mechanism for fostering otherness.

“Music allows us to experience other narratives and explore other worlds that we sometimes don't know. Feelings that we haven't felt so much—music brings that to you, makes you feel it in some way. It's like thinking about a feeling that isn't mine, but that the song makes me feel and transports me to. I believe this exercise in otherness that music brings was very important.” F2‐M‐U1

The mutual exploration of diverse emotional realities was easier when participants were emotionally connected. Participants felt that the emotional exchange was reciprocal during the sessions, and they invested time and effort to engage with each other. To see their colleagues truly expressing themselves and welcoming others to do the same made some students feel confident to share their authentic selves. For example, one facilitator reported that, following a session, a student initially hesitated to express his emotions and remained in silence. However, when a colleague described her interpretation of the sadness in the lyrics and openly cried, the first student put down the shield and he also cried. In general, students valued the rare opportunity of exchanging their authentic emotions within an academic culture that is seldom receptive to these experiences. They considered this opportunity “a gift” ‐ a source of support and confidence that encouraged them to connect to each other.

“I thought it was very interesting that we were able to share such different ideas with the same music and the same situation. So, I think that's a gift to see different experiences, to see different interpretations. I find it very interesting to be able to see in the other person what I'm not seeing. I find that very captivating.” S14‐F‐U2

Stimulated by curiosity about the other and the desire for meaningful and authentic interactions, most students recognized that increasing their emotional awareness could enhance their ability to understand the other. Music facilitated access to deeper emotional connections not readily attainable through ordinary verbal communication.

“And then music comes along, bringing sensations, broadening horizons, bringing emotions that go beyond the limits of words. I think they both [music and medicine] manage to transport us to a higher level of emotion and that it's possible to think and feel at the same time. Music can transport you to that great spectacle of being together with a new, different person.” S19‐M‐U3

### 3.3 Providing opportunities to engage with EmtR strategies

As students reflected on their emotions during the activity, they recognized mechanisms by which emotions influence professional practice. By becoming aware that their emotions could influence their professional attitudes, most students demonstrated interest in exploring the consequences of their emotional states. They understood that, depending on the context, clinical situation and persons involved, preexistent emotions could facilitate or hamper the communication and connection with patients, peers and colleagues.

“Something I've learned a lot is to have the notion that the emotion we feel that day, at that moment, the things we have been carrying will affect how we see that process of care or what the patient is going to transmit to us.” S10‐F‐U2

Music and discussion of clinical vignettes functioned as structured opportunities to experience EmtR mechanisms. Some students actively engaged with and applied EmtR mechanisms ‐ they understood that EmtR can alter the trajectory of complex emotions. By discussing the possible outcomes for clinical vignettes and also by reflecting on how composers and performers used examples of EmtR in song lyrics, students observed the role of different EmtR mechanisms in the management of situations charged with emotions. This opportunity to recognize that EmtR can influence outcomes in medical practice enriched the subsequent discussions surrounding clinical vignettes and afforded students the opportunity to anticipate medical‐related emotions.

“It was important, because I've always been afraid of how I'm going to act, that I'm not going to succeed. You have many questions, and realizing that this is a training process, where we stop, look at the situation and reason, and it's okay to feel! But what are we going to do with it, you know? And having thought about certain situations in advance to be able to act better, not excluding emotion, was very important. I think I will take it with me. I think I've already taken it with me.” S21‐F‐U3

The interviewed facilitators reported drawing parallels between the emotions evoked by music and the ones evoked during the management of the dynamic and sometimes unpredictable situations in medical practice. Facilitators believe that exploring differences and similarities in such emotional processes may deepen students' awareness of the role of emotions in doctor‐patient interactions and highlight the need for continuous emotional development to endure the high emotional demands of the medical profession.

“I really liked the examples (in the vignettes) of how emotions can be present in our (clinical) practice to facilitate the connection with patients and promote better care. Being there (with the patient) as a person—not just someone who is going to heal, but a caregiver who listens, feels alongside the patient, and allows themselves to be touched, just as we allowed ourselves to be touched by music. I think that's it.” F1‐F‐U1

The facilitators also conducted discussions on inefficient EmtR mechanisms that should be avoided in medicine, due to their deleterious impact on both patients and doctors. For instance, in a vignette where a doctor failed to perform a tracheal intubation due to anatomical variations in the patient's airway, some students experienced shame and guilt, envisioning themselves embarrassed by the technical failure ‐ a harmful EmtR mechanism of attentional deployment: emotional rumination. Others said that doctors often avoid emotions after a technical failure, believing that emotional detachment is necessary ‐ a deleterious EmtR mechanism of response modulation: emotional suppression. However, some students felt encouraged to reflect on and recognize the uncertainties inherent to clinical practice ‐ a desirable and different EmtR mechanism: cognitive reappraisal. Facilitators used the discussion about different EmtR mechanisms to invite students to reflect on how important it is to develop awareness of how our emotional status influences our medical practice and how EmtR mechanisms can be used in favour or against better emotional coping. Some students shared that this in‐depth reflection on the role of emotions in medical practice may have allowed them to navigate and make sense of their emotional reactions and understand how important it is to develop EmtR mechanisms consciously to become a competent doctor.

“And, as we learned, this process of reappraising, of changing the emotional trajectory, all of that was very important for me in the sense of not allowing myself to be dominated by monsters that I had hidden and thrown under the carpet. So, I need to face this tough reality, and that means maturing, growing up and becoming stronger through the difficulty, not omitting myself. I think that was the most important thing for me.” S19‐M‐U3

### 3.4 Naturalizing emotions in medicine

Some students recalled that their motivation to become a doctor comes from an emotional state that involves the joy and fulfilment resultant from caring about others, wanting to help, connecting with patients and contributing to their well‐being. However, these students perceived that these emotions had been progressively neglected throughout their journey as medical students. They attributed this emotional negligence to two elements of the hidden curriculum in medicine: first, the medical curriculum offers a predominance of technical activities with little space dedicated to nurturing students' emotional development. Second, they perceived that there is an implicit assumption that to be a good doctor, you have to suppress your emotions. As a result of this process, they became less aware of their and others' emotions as they went through the medical training.

“It's a perpetuated culture that you can't feel anything, that you have to be there without any feeling, an analytical coldness that, in fact, I don't think exists. It doesn't exist; it's a lie.” S18‐F‐U3

Some students reported that they felt reconnected with the idea of becoming doctors by rediscovering the pleasure of sharing emotions, connecting with patients and their colleagues and understanding the mechanisms of EmtR. They felt better prepared to face the emotional challenges of medicine. For instance, in one session, a student expressed anger towards a patient who did not follow the doctor's prescription for taking a medicine. By paying more attention to her own emotions, she understood that this anger was actually frustration about feeling impotent by not being able to help. Moreover, by shifting her attention to the patient's emotions, she realized that the patient was demanding emotional support and encouragement to start the lifestyle changes and possibly adhere to the prescription in the future. Empowered by this reconnection with their emotional foundations, some students started to build a bridge linking their personal and professional lives.

“And thinking about the profession itself, I think there's no way to dissociate the two things (personal and professional), because we're human beings first and foremost, but it brought me even more of a sense of purpose, of knowing that there's this view (valuing emotions) in medicine, that there's this view in the profession, that it's not in vain to study about these things (emotions).” S13‐F‐U2

Facilitators reported inviting students to reflect on professional performances where emotions were expressed openly and authentically. For instance, one facilitator shared a situation in which a doctor, visibly moved to tears, demonstrated compassion, maintained presence and nurtured connection while breaking bad news to a patient about the diagnosis of metastatic cancer. This facilitator believes that sharing such experiences invites students to acknowledge the inevitability of emotional experiences in their future careers and that their emotional states could lead to deeper and true connections with their patients.

“As doctors, we can't just stick to theory. We need to go into practice and experience our emotions. The emotions that are so important and that will help us create connections with our patients.” S9‐F‐U2

Most participants believe that through these processes, music listening and structured reflection operated as mechanisms that helped students to naturalize emotions in medicine. They reflected on how physicians often find themselves navigating the balance between their personal values, beliefs and experiences, and the rigorous demands and responsibilities of their professional roles. This intersection shapes not only how they approach patient care and medical decision‐making but also influences their overall well‐being and sense of fulfilment with the profession. Several students reported that they were adopting new behaviours during their clinical rotations. They started to consider their emotions, and the emotions of patients, as an important part of the medical encounter and reported experiencing better connections with their patients, as a consequence of applying what they learned about EmtR. They also mentioned a feeling of rescuing the principles that motivated them to choose medicine as a profession.

“Emotion is a human thing. And since emotion is a human thing, it will appear in medicine. In medicine, there will be emotional moments that I will have to deal with. This is very clear to me. I'm a human being, human beings have emotions, and since I'm going to be working with human beings, I'm going to be working with emotions.” F4‐M‐U3

## 4 DISCUSSION

Our study explores the mechanisms through which a music‐based pedagogical activity nurtures medical students' emotional connections and promotes the understanding and utilization of EmtR mechanisms. Our results suggest that music listening and guided reflection operated as pedagogical processes through which students revisited emotional biographies. They felt encouraged to explore the emotional dimensions of becoming a doctor and practicing medicine. It seems they freed their imagination and engaged with the complex emotional dimensions presented in the clinical vignettes and reflected on how the artists used examples of EmtR in their songs. This unconstrained imagination may have initiated a process of “emotion simulation”, enabling students to practice EmtR strategies within a safe environment.^37^, ^38^ We believe that this process of “emotion simulation” facilitated a comprehensive understanding of the role of emotions in medical practice and allowed students to recognize emotions as a natural element of their careers.

In the following paragraphs, we will draw parallels between our results and three theoretical concepts: sensible cognition, “re‐creation as creation” and music‐evoked autobiographical memories (MEAMs). These concepts did not guide our data collection or analysis; rather, they serve as interpretative lenses to deepen our reflection on the results. This post hoc engagement with theory aligns with our inductive approach, allowing the data to speak before positioning it within existing frameworks.^39^ Given the limited exploration of how subjective domains such as art and music may enrich medical students' emotional development, mobilizing theory at this stage extends the interpretive reach of our study and anchors it in broader scholarly conversations.^40^

In his seminal work on *aesthetics*, the German philosopher Alexander Baumgarten defined the concept of sensible cognition as the process by which sensory perception contributes to knowledge production by integrating sensory experiences with intellectual processes.^41^ Baumgarten argued that art modalities, such as music, play a critical role in developing sensible cognition by generating understandings beyond words, helping us to make sense of the world.^42^ In our study, listening to music created an opportunity for students to imagine, visualize and reflect on various emotional responses. By enabling such abstractions, music may have broadened perspectives and facilitated a deep exploration of emotions, possibly contributing to expanding students' understanding about the role of emotions in their personal and professional lives.^43^ We believe that music listening functioned as a mechanism for bridging sensory and cognitive domains. We concluded that integrating sensory and intellectual experiences in educational settings may foster emotional awareness and development by coalescing perception, reflection and imagination.

Our results suggest that the invitation to exercise their imagination during music listening allowed students to amplify their capacity to make sense of their emotional experiences. This meaning‐making process aligns with Dilthey's theory of “re‐creation as creation,” which posits that understanding is achieved through re‐experiencing and interpreting one's own and others' emotions.^44^ We believe that the re‐creation occurred when music engaged students in an active process of imagining emotional situations, facilitating a type of comprehension that is rooted in the direct, experiential engagement with emotions. By imagining these situations, students could reflect on their own emotions while sharing and exploring the emotional reactions of other participants, fostering a collective construction of knowledge. This aligns with previous works that have observed how co‐constructive learning environments may facilitate emotional connection and enable students to explore a broader range of emotional situations, opening space for the construction of new meanings.^45^, ^46^ Such complex meaning‐making processes are vital for medical education, as they help students to expand their perspectives, understand the emotional dimension of patients and provide opportunities to integrate knowledge from outside the realm of natural sciences.^47^, ^48^, ^49^, ^50^ In the context of experiencing, imagining and making sense of these emotions, students may create simulated scenarios in which they alter the trajectory of more complex and challenging emotions, re‐interpreting these emotions through the lens of EmtR mechanisms.

The link between EmtR and imagination is deeply intertwined, as both processes involve the cognitive manipulation of emotional experiences. Imagination allows individuals to mentally simulate scenarios that evoke emotional responses, providing a safe space to explore and manage emotions.^51^ For instance, one of the key mechanisms of EmtR is cognitive reappraisal, which involves changing the way one thinks about a situation to alter its emotional impact. Imagination plays a crucial role in this process by allowing individuals to visualize alternative interpretations or outcomes of events, reshaping their emotional responses.^38^ Listening to music may also contribute to cognitive reappraisal by providing new perspectives on personal experiences and helping individuals to reframe their emotions.^52^ Such opportunities to practice EmtR may offer students the chance to realize that EmtR is not just about managing emotions in real‐time but also involves transforming “negative” emotions into opportunities for improvement. Music may support this EmtR transformation process by enabling individuals to revisit the past and identify these tough emotions, serving as a powerful contextual cue that evokes autobiographical memories.^27^

Music‐evoked autobiographical memories (MEAMs) refer to the phenomenon by which specific songs trigger the recall of self‐defining moments in one's past.^25^ By retrieving MEAMs, students may engage in reflection and self‐exploration, revisiting significant past moments that highlighted the emotions, values and goals important to them at different stages of their lives. This reflection may lead to a deeper understanding of who they are and how their experiences have shaped them. By recalling past experiences and emotional states, students can identify themes that reveal their authentic passions and purposes, providing clarity on personal and professional goals while enabling alignment of current actions with long‐held values.

With their sensitivity heightened by sensible cognition, their ability to reinterpret their emotional experiences amplified by re‐creation as creation, and strengthened by EmtR mechanisms, students can seize the opportunity provided by the retrieval of their autobiographical memories to integrate their professional goals and authentic values with their re‐shaped emotional selves. This integration may bring a renewed sense of purpose to their journey of becoming a doctor. For several students, the music‐based pedagogical activity rescued their inner drive to embrace the medical profession.

## 5 PRACTICAL IMPLICATIONS

Our findings support previous research demonstrating that art‐based pedagogies, such as music, can significantly enhance the personal, professional, social, cognitive and emotional development of medical students.^30^, ^53^ Music provides numerous examples of EmtR mechanisms through its melodies, harmonies, rhythms and lyrics, making it a powerful medium for disseminating EmtR theory and fostering the emotional development of medical students.^54^, ^55^ Integrating art‐based pedagogies is a crucial step toward strengthening the medical curriculum, with the potential to counteract the growing trend of emotional detachment and mental suffering in medical schools.^53^, ^56^ EmtR mechanisms contribute to this effort by expanding the potential of art‐based pedagogies to support the recognition, expression and regulation of difficult emotions in safe and structured ways.^57^, ^58^, ^59^

However, to effectively implement such pedagogical activities, it is crucial to develop faculty development programs that equip educators with the necessary skills to put into practice the mechanisms identified in our study and the capacity to discuss and model EmtR strategies.^60^ Such programs could incorporate EmtR principles and enable educators to recognize and address emotional dynamics in both medical practice and educational settings. Equipping medical educators to embrace the emotional dimension of their practices may counteract the hidden curriculum, which often discourages emotional expression, and contribute to the development of a more emotionally attuned medical workforce.

## 6 STRENGTHS AND LIMITATIONS

One of the strengths of our study is the inclusion of both students and facilitators' perspectives in the reflexive thematic analysis, which enabled a comprehensive understanding of how different participants engaged with the pedagogical mechanisms observed and how they fostered their emotional development. Another strength is the embedding of our results on solid theoretical principles that may guide the adaptation of this activity to diverse contexts. We believe that this pedagogical innovation holds potential for application across different stages of medical training, in postgraduate education and even beyond the health professions—given that emotional development is a foundational element of personal development, regardless of professional background.

Our study has several limitations. One is that participation in the study was voluntary, meaning that both students and facilitators self‐selected into the study. As such, participants were likely already interested in the topic of emotional development, which may have influenced their level of engagement and the overall positive outcomes observed. In broader implementation contexts where participation is mandatory or interest varies, similar results may not be as easily replicated.

Another limitation of our study was the use of two different formats for conducting the interviews: in‐person at Site 1 (between February and March 2020) and remotely via Zoom at Sites 2 and 3 (between June and August 2021), due to restrictions imposed by the COVID‐19 pandemic. Although both formats followed the same semi‐structured guide, differences in interpersonal dynamics may have influenced the depth of interaction and participants' engagement during the interviews.

We did not address how students' social and cultural/ethnic backgrounds influenced their emotional development during the activity. Our analysis also did not evaluate the impact of the different musical styles chosen by the participants, nor did we evaluate the themes of the songs and their association with the students' emotional development or other outcomes. Finally, our research group firmly believes in the importance of supporting medical students' emotional development, and this belief influenced not only the design of the educational intervention but also the process of data analysis.

## 7 CONCLUSION

Pink Floyd's classic song “Comfortably numb” from the album and movie “The Wall” translates into a melancholic melody a sad narrative of a musician who feels emotionally detached from the world around him.^61^ This emotional detachment can resonate deeply with those experiencing intense professional and personal pressures, such as medical students, when engaging with suffering and death. Although they have the privilege of helping others by being part of their healing process, they often hide their own emotions and become numb in a state of emotional suppression.^15^

By embracing the transformative power of music and EmtR, we bring a potential innovation to the medical curriculum, aiming to equip future doctors with the tools to foster healthier work environments and improve patient care. Our study aimed to highlight the importance of emotional development, tearing down the walls that isolate students from their emotions.

## AUTHOR CONTRIBUTIONS

**Marcelo B. S. Rivas:** Conceptualization; methodology; data curation; investigation; validation; formal analysis; visualization; project administration; resources; writing—original draft; writing—review and editing. **Agnes F. P. Cruvinel:** Writing—original draft; writing—review and editing; methodology; conceptualization; formal analysis. **Daniele P. Sacardo:** Conceptualization; writing—original draft; methodology; writing—review and editing; formal analysis. **Daniel U. C. Schubert:** Writing—original draft; methodology; visualization; writing—review and editing; data curation; investigation; resources. **Mariana Bteshe:** Writing—original draft; methodology; visualization; writing—review and editing; data curation; investigation; conceptualization; validation; supervision; formal analysis. **Marco A. Carvalho‐Filho:** Conceptualization; investigation; writing—original draft; writing—review and editing; formal analysis; project administration; supervision; software; methodology; data curation; validation; visualization.

## CONFLICT OF INTEREST STATEMENT

The authors declare no conflicts of interest.

## ETHICS STATEMENT

Granted by the research ethics committee on human beings from the Faculty of Medical Sciences of the Campinas State University (STUDY#4.605.559); the School of Medicine of Rio de Janeiro State University (STUDY#3.276.206); and the School of Medicine of Federal University of Fronteira Sul (STUDY#4.626.426).

## PREVIOUS PRESENTATIONS

The authors presented portions of this work at the Association for Medical Education in Europe (AMEE) Conference in Glasgow, Scotland, August 2023.

## Data Availability

The data that support the findings of this study are available from the corresponding author upon reasonable request.
